# PTBP1 Is Required for Embryonic Development before Gastrulation

**DOI:** 10.1371/journal.pone.0016992

**Published:** 2011-02-17

**Authors:** Jakob Suckale, Olivia Wendling, Jimmy Masjkur, Melanie Jäger, Carla Münster, Konstantinos Anastassiadis, A. Francis Stewart, Michele Solimena

**Affiliations:** 1 Molecular Diabetology, Paul Langerhans Institute Dresden, School of Medicine and University Clinic ‘Carl Gustav Carus,’ Dresden University of Technology, Dresden, Germany; 2 Department of Functional Genomics, IGBMC (Institut de Génétique et de Biologie Moléculaire et Cellulaire) & ICS (Institut Clinique de la Souris), Illkirch, France; 3 Center for Regenerative Therapies Dresden, BioInnovationsZentrum Dresden University of Technology, Dresden, Germany; 4 Genomics, BioInnovationsZentrum, Dresden University of Technology, Dresden, Germany; 5 Max Planck Institute of Molecular Cell Biology and Genetics, Dresden, Germany; Wellcome Trust Centre for Stem Cell Research, United Kingdom

## Abstract

Polypyrimidine-tract binding protein 1 (PTBP1) is an important cellular regulator of messenger RNAs influencing the alternative splicing profile of a cell as well as its mRNA stability, location and translation. In addition, it is diverted by some viruses to facilitate their replication. Here, we used a novel *PTBP1* knockout mouse to analyse the tissue expression pattern of *PTBP1* as well as the effect of its complete removal during development. We found evidence of strong *PTBP1* expression in embryonic stem cells and throughout embryonic development, especially in the developing brain and spinal cord, the olfactory and auditory systems, the heart, the liver, the kidney, the brown fat and cartilage primordia. This widespread distribution points towards a role of *PTBP1* during embryonic development. Homozygous offspring, identified by PCR and immunofluorescence, were able to implant but were arrested or retarded in growth. At day 7.5 of embryonic development (E7.5) the null mutants were about 5x smaller than the control littermates and the gap in body size widened with time. At mid-gestation, all homozygous embryos were resorbed/degraded. No homozygous mice were genotyped at E12 and the age of weaning. Embryos lacking *PTBP1* did not display differentiation into the 3 germ layers and cavitation of the epiblast, which are hallmarks of gastrulation. In addition, homozygous mutants displayed malformed ectoplacental cones and yolk sacs, both early supportive structure of the embryo proper. We conclude that *PTBP1* is not required for the earliest isovolumetric divisions and differentiation steps of the zygote up to the formation of the blastocyst. However, further post-implantation development requires *PTBP1* and stalls in homozygous null animals with a phenotype of dramatically reduced size and aberration in embryonic and extra-embryonic structures.

## Introduction

Mouse embryonic development lasts about 3 weeks in total. During the first days after fertilization the embryo does not grow significantly in size, remaining at around 0.1 mm in diameter, and differentiates only into 2 tissue types, the outside trophoblast and the inner cell mass. The number of embryonic cells grows exponentially to ∼70 at the time of implantation with concomitant decrease in volume per cell.

Implantation at about day 4.5 of embryonic development (E4.5) [1] marks a change in pace. After this point, the volume of the embryo increases exponentially from ∼0.2 mm in length at E4.5 to 0.7–0.8 at E7.5. In the process embryonic tissues rapidly reshape and differentiate. At E5.5, the compact embryo forms a cavity and turns into the egg cylinder. A day later at E6.5, gastrulation begins with the formation of the primitive streak in the posterior region of the epiblast. Briefly afterwards the 3 germ layers, ectoderm, mesoderm and definitive endoderm, are formed [2]. This marks the beginning of major differentiation events.

Before implantation the embryo depends mostly on its own reserves, while implantation represents a transition from internal to external supply. The embryo and maternal cells form early supportive structures like the yolk sac, the ectoplacental cone and eventually the placenta, which nourishes the embryo and induces adaptation in maternal cells [3]. Similarly, gene expression switches from preloaded maternal to embryonic mRNAs. In mice this maternal to zygotic transition lies relatively early at around the 2-cell stage or E1. RNA-binding proteins, like PTBP1, influence both splicing of pre-mRNAs as well as their stability and translation into protein.

PTBP1 (also known under the non-standard names PTB and hnRNP I) is a nucleic acid binding protein, mainly known for its role in multiple aspects of mRNA life cycle and function. These include regulation of splicing [4]–[9], 3′-end processing [10], [11], internal ribosomal entry site-mediated translation [12]–[14], localization [15], [16] as well as stability and translation [17]–[21]. PTBP1 may also bind DNA [22] and act as a transcription factor [23], [24].

*PTBP1* is a member of a larger family of 4 currently known genes in mammals: *PTBP1* [25], *PTBP2* [26], *ROD1* [27] and *smPTB* [28]. Mammalian *PTBP1* is broadly expressed and the best studied gene in the group. *PTBP2* (nPTB) is synthesised especially by neuronal cell types. *ROD1* has been detected in rat hematopoietic cells, while *smPTB* is expressed in smooth muscle. The *Drosophila* orthologue of *PTBP1* is *hephaestus*, which in the oocyte is part of the oskar mRNA-localising complex where it represses its translation [17]. Later, during fly embryo development it is expressed in mesodermal and neuronal lineages as well as the imaginal discs and the adult germline [29].

According to structural data [30]–[33] PTBP1 contains 4 RNA-binding domains. These domains belong to the RNA recognition motif (RRM) family, one of the most abundant nucleic acid-binding domains [34]. RRMs of different proteins bind 2–8 nucleotides with the RRMs of PTBP1 binding 3–5 with slightly different sequence preference [35]. Multiple RRMs in a single protein increase overall affinity and specificity.

Members of the PTBP family cross-regulate each other. Besides regulating its own levels by exon suppression leading to nonsense-mediated decay [4], PTBP1 suppresses PTBP2 via a similar mechanism [5], [6]. When PTBP1 levels are reduced, PTBP2 levels are often upregulated. This hand-over causes a broad shift in splicing and is critical for neuronal differentiation [5], [6]. Reduction of PTBP1 by RNAi in HeLa cells did not significantly changeprotein levels except for the release of PTBP2 suppression, which demonstrates the redundancy between these proteins. Only when both were reduced did protein levels for several genes change, including the release of ROD1 suppression [7].

In order to investigate the function of PTBP1 in a complex organism, we set out to generate a mouse multi-functional allele. In this manuscript we report the developmental expression pattern of *PTBP1* as detected with the reporter gene introduced into this allele as well as the effect of its systemic knockout.

## Results

### A novel multi-purpose allele for *PTBP1*

**Figure 1:**
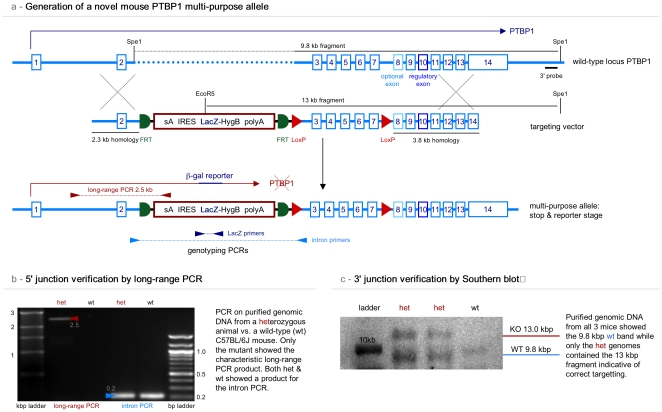
Generation of a mouse containing a novel multipurpose *PTBP1* allele. We generated a multipurpose *PTBP1* allele and targeted it into mice. A) The wild-type locus (blue) was modified with the insertion of a stop/detection cassette (red) into embryonic stem cells. The cassette can be flipped out using an FLP recombinase construct or strain. In addition, exons 3 to 7 have been framed with loxP sites for conditional removal by a Cre recombinase. The diagram indicates the Southern strategy as well as the location of the primers for validation of the construct and genotyping. B) shows the results of the long-range PCR to verify the 5′ junction resulting from the homologous recombination of the targeting vector with the wild-type *PTBP1* locus. C) Southern blot to verify the correct 3′ junction in the mutant allele.

Using Red/ET homologous recombination [36] we generated a novel *PTBP1* allele (Figure 1, Figure S1), which in its original state causes a systemic knockout and produces bacterial β-galactosidase (*LacZ*) for detection of expression from the *PTBP1* locus. We verified the efficiency of the transcriptional block using RT PCR (Figure S2). The stop/detection cassette can be removed with FLP recombinase leading to an allele indistinguishable from the wild-type except for the presence of loxP sites flanking exons 3 to 7. In the last stage of the multi-purpose allele, this group of exons can be removed by Cre recombination, leading to a premature stop codon and a null mutation of PTBP1.

### Broad expression of *PTBP1* during gestation

**Figure 2:**
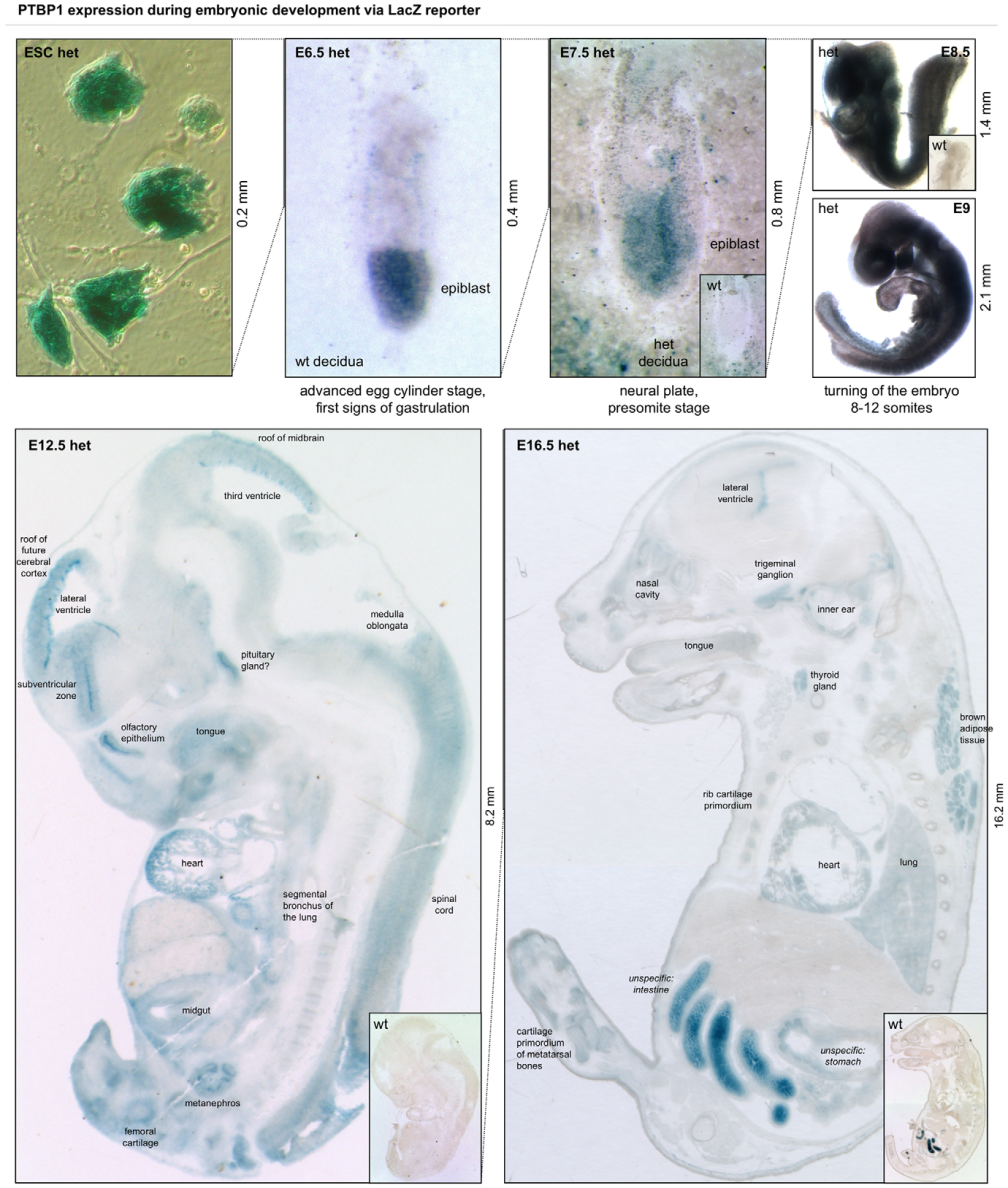
*PTBP1* is expressed throughout embryonic development. The LacZ reporter shown in Figure 1 was used here as a proxy for *PTBP1* promoter activity in heterozygous embryonic stem cells (ESCs) and embryos at different stages of development. *PTBP1* expression was high in ESCs. In embryos 6.5 days after fertilisation (E6.5), *PTBP1* was expressed especially in the epiblast but also in the visceral endoderm surrounding the embryo. The expression pattern was similar at E7.5 where the activation of maternal *PTBP1* was seen in the decidua surrounding the tip of the embryo. No LacZ staining was observed in the wild-type (wt) littermate (small inlet). The reporter was expressed in almost all cells at E8.5 and in most cells at E9, when only the areas at the roof of the future head, the primitive ventricle of the heart, and some structures in the tail appeared to express little or no *PTBP1*. In E12.5 embryos most developing tissues and organs expressed the reporter, most notably the cells lining the ventricles of the brain, the spinal cord, the pituitary and the heart. Similar organs were stained in E16.5 embryos and in addition the nasal cavities, the inner ear and the brown fat pad on the back of the embryo. *PTBP1* was high in the ventral rib cartilage but much lower in the dorsal rib bones that already showed a central cavity. While at E12.5 there was no unspecific staining, at E16.5 the intestine and to a lesser degree the stomach displayed an X-gal signal not due to the *PTBP1* reporter. The signal result from endogenous β-galactosidase. See Figure S3 for a whole-mount LacZ at E12 and E16.

In order to gauge the importance of PTBP1 in embryonic development, we performed X-gal staining of heterozygous embryonic stem cells (ESCs) and heterozygous whole embryos at equal time intervals between implantation at E4-4.5 and birth at E20-21 (Figure 2). *PTBP1* displayed a striking expression pattern in E6.5 and E7.5 basically labelling the epiblast. In E6.5, we observed *PTBP1* reporter in visceral endoderm but not in yolk sac mesoderm, chorionic ectoderm or the ectoplacental cone. Heterozygous but not wild-type decidua expresses *PTBP1* in the area surrounding the epiblast. Since X-gal only penetrates 1–2 mm into tissue, we performed X-gal staining on cryosections in addition to whole mount preparations for E12.5 and E16.5. Accordingly, the LacZ signal in the skin was very strong in whole mount preparations (Figure S3), but barely detectable in the cryosections (Figure 2), which instead revealed strong *PTBP1* expression in developing internal organs and tissues such the brain cortex and subventricular zone, where neuronal precursors are found, the trigeminal ganglion, the nasal cavity, the inner ear, the medulla oblongata, the spinal cord, the thyroid, the heart, the lung, the kidney, possibly the pituitary and several cartilage primordia but not more ossified structures (see especially the E16.5 rib sections in Figure 2). *PTBP1* expression became more restricted with increasing embryonic age. While the E8.5 embryo was almost completely stained by X-gal, the signal became more differentiated in E12.5 and E16.5. We conclude that *PTBP1* is strongly and broadly expressed during the entire gestational period and in the cultured embryonic stem cells used to generate this knockout mouse model.

### PTBP1 null embryos implant but are severely growth-retarded

To gain insight into the function of PTBP1, we analyzed the progeny of mice heterozygous for the *PTBP1* multipurpose allele. We noted that the average heterozygous/heterozygous litter of 7.0±1.7 standard deviation was smaller than a heterozygous/wild-type cross with 8.2+/−2.2. Genotyping at weaning revealed the absence of homozygous offsprings and a 0.57 ratio between wild and heterozygous (Table 1). This data indicated that *PTBP1* null mutants are embryonically lethal and thereby that this gene is essential for development.

**Table 1:** Survival of PTBP1 null mutants.

		genotype			ratio PCR		ratio IF
age	method	wt	het	hom	wt/het	hom/het	hom/het+wt
E6.5	IF	19		**5**			0.26
E7.5	PCR	5	12	**5**	0.42	0.42	
E7.5	IF	3		**3**			1.00
E8.5	IF	7		**2**			0.29
E12.5	PCR	8	17	**0**	0.47	n/a	
E16.5	PCR	1	3	**0**	0.33	n/a	
3 weeks	PCR	25	44	**0**	0.57	n/a	
				expect:	0.50	0.50	0.33

The table summarises the genotype of embryos during gestation as determined by PCR or immunofluorescence (IF), the latter of which cannot distinguish between wild-type (wt) and heterozygous (het). The last viable homozygous (hom) embryos were detected at day 8.5 of embryonic development (E8.5). No homozygous mutants were found at E12.5 or later. Genotype ratios observed and expected are shown on the right. While they are close to the expected Mendelian ratio for experiment with several litters, random variations and deviation from the expected ratios are larger in single litter experiments.

**Figure 3:**
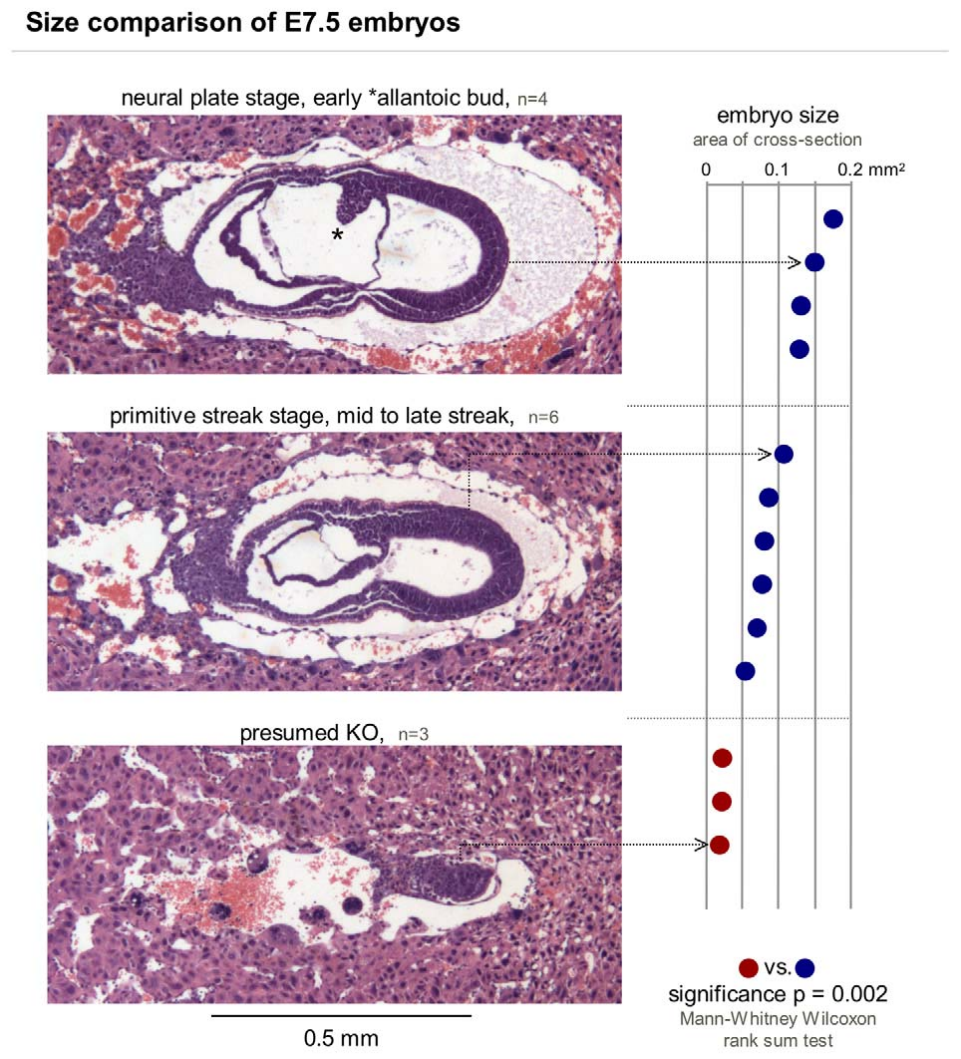
Embryos from *PTBP1* KO heterozygous parents fall into 2 size categories. Hematoxylin and eosin stained decidual sections of 2 litters were analysed for surface area and structural organisation. Embryos were grouped according to their developmental stage (left) with the furthest developed having reached neural plate stage but most embryos being at the primitive streak stage. The surface area of the embryos in the middle section was measured with the Fiji image software and ranked according to size, which turned out as equivalent to stage (right). Presumed knockouts (red) were significantly smaller than wild-type or heterozygous controls and present in a roughly Mendelian ratio of 3∶10 (ratio 0.3 vs. the expected 0.25). The size of the small embryos might have even been overestimated since it was harder to recognise the embryo in those implantations. We also encountered 2 decidua with no visible embryonic structures.

To establish at which stage the lack of PTBP1 stalls embryonic development we isolated embryos working backward from E12.5. At this time 2 types of implantations were visible: small degraded and normally sized. Going further backward in development we were able to observe small implantations at E6.5, E7.5, and E8.5 (Table 1), the last time point examined at which small embryos still showed cellular structure and were not yet degraded. At E7.5, we quantified the area of the largest embryo cross-section. The small embryos were on average 0.02 mm^2^, significantly smaller than the large embryos with 0.11 mm^2^ (Figure 3). In 2 litters we observed 3 small embryos and 10 large embryos (ratio 0.3) and 2 empty deciduas.

**Figure 4:**
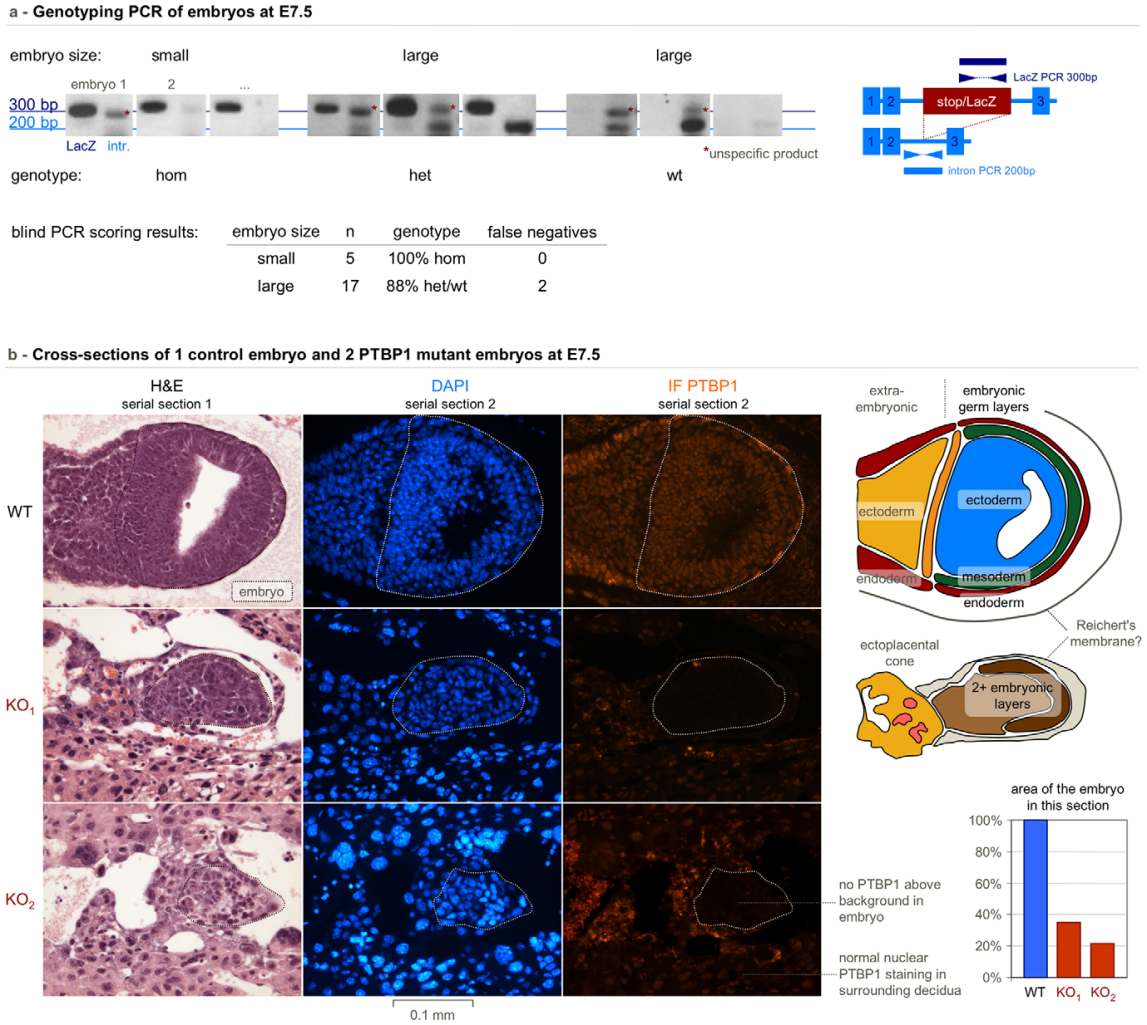
PTBP1 knockout embryos implant but are severely growth-retarded. The small category of embryos from a heterozygous intercross were identified by PCR and immunofluorescence as PTBP1 knockout embryos. A) PCR products for the stop cassette and *PTBP1* intron 2 were separated on agarose gels and scored blindly. 5/5 small embryos were genotyped as homozygous. 15/17 large control embryos were genotyped as heterozygous or wild-type with 2 false negatives due to the less efficient intron PCR. B) Serial paraffin sections of E7.5 embryos were stained with hematoxylin and eosin (left column) or labelled with DAPI (middle column) and the PTBP1 antibody (right column). The top row shows a large embryo (in an oblique section) with strong nuclear PTBP1 staining of the embryo proper (dashed line) and the surrounding tissue. Both small embryos were characterised by a lack of the nuclear PTBP1 signal while showing nuclear PTBP1 in surrounding, most likely maternal cells. Interpretative diagrams and a quantification of the embryo section area are shown on the right.

In order to ascertain the nature of the small implantations, we isolated embryos at E7.5, carefully avoiding contamination from the maternal decidua which would have biased the resulting genotype towards heterozygous. We analysed 5 small and 17 large embryos by PCR. All 5 small embryos were genotyped as homozygous for the *PTBP1* knockout allele, 15 large embryos were either wild-type or heterozygous, with 2 large embryos being misclassified as homozygous probably due to problems in the intron PCR (Figure 4 and Table 1).

We confirmed the hypothesis that small embryos are homozygous by immunofluorescence staining with a PTBP1 antibody (Figure 4). All small embryos at E7.5 showed only background level of PTBP1 signal from the embryonic tissue while showing normal nuclear fluorescence from surrounding maternal heterozygous decidual cells. There was, however, unspecific staining in the yolk sac membrane due to the use of a mouse monoclonal antibody. Together with the PCR data this establishes that homozygous embryos are severely growth retarded compared to their littermates.

### Null embryos are not able to begin cavitation or properly form the yolk sac and the ectoplacental cone

**Figure 5:**
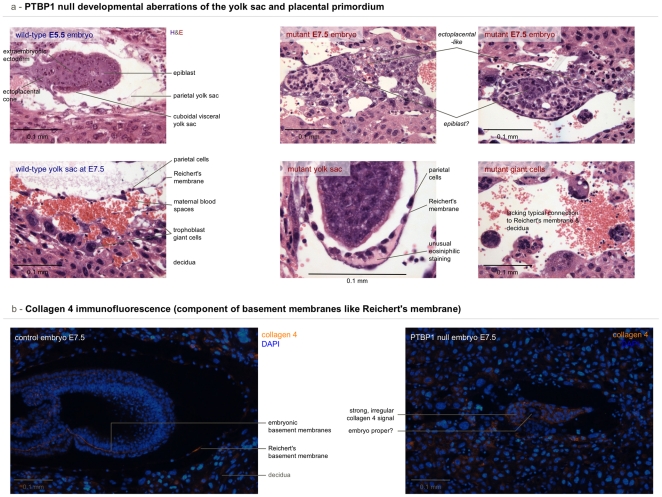
PTBP1 null embryos show defects in yolk sac & placenta development. This figures compares yolk sac and placental structures in knockout and control embryos. A) Hematoxylin and eosin-stained paraffin sections of an E5.5 wild-type embryo, chosen for its comparable size to the null mutants, and an E7.5 yolk sac (left) juxtaposed with mutant E7.5 embryos and mutant yolk sacs, respectively (right). Mutants lack the typical structure of a normal embryo at this stage. If discernable at all they lack a typical yolk sac with a thin Reichert's membrane in close proximity to trophoblast giant cells and the decidua. B) Immunofluorescence for collagen 4 on a section of a control E7.5 embryo is shown next to a similar image from a homozygous embryo. The control embryo shows collagen 4 signal from several basement membranes including the Reichert's membrane of the yolk sac. The null mutant, on the other hand, shows an unusually extended area high in collagen 4, not resembling a membrane. Separate channels for the collagen immunomicroscopy can be seen in Figure S4.

A wild-type embryo at E7.5 has formed an ectoplacental cone invaded by maternal blood [3] and has surrounded itself with a yolk sac (Figure 5a). The latter structure is cooperatively build by parietal endoderm forming a thick basement membrane (the Reichert's membrane) and trophoblast giant cells on the embryo side, as well as maternal blood and decidual cells on the outside [37]. In many homozygous mutant embryos the yolk sac was not clearly separated from the epiblast. Also, the Reichert's membrane was not properly formed and contained unusual quantities of eosinophilic material (Figure 5a). Similarly, the ectoplacental cone did not properly form, often lacking the characteristic connection of decidual cells, giant cell, the Reichert's membrane and parietal cells. A typical embryo is enveloped in visceral endodermal cells, which are subdivided into cuboidal cells with characteristic apical vacuoles and squamous cells that differentiate later. Even in the most advanced homozygous mutant at E7.5, the squamous endodermal cells were not visible showing a delay in the formation of the visceral yolk sac.

To look in more detail at the molecular composition of control and mutant membranes, we performed immunofluorescence on embryo cryosections (Figure 5b) for collagen 4, which is an abundant and essential structural component of the basement membrane [38]. Normal embryos (wild-type or heterozygous) showed a strong collagen 4 signal from the Reichert's membrane as well as the basement membrane underlying the visceral endoderm. On the other hand, embryos lacking *PTBP1* had a massively increased area high in collagen surrounding a small spherical area containing little or no collagen, which might represent the embryo proper.

We speculate that since the developing placenta and the growing yolk sac nourish the embryo and are involved in signalling processes, null embryos may be starved and insufficiently stimulated to grow and develop.

### PTBP1 knockouts do not complete stages of early development including cavitation and gastrulation

In normal embryos starting at ∼E5, the epiblast hollows to form a central cavity which at E7.5 is subdivided into the ectoplacental, the exocoelomic, and the amniotic cavity. Cavitation becomes easily discernible in sections at E6. We never observed cavitation in thin sections of a dozen homozygous *PTBP1* null embryos at E7.5 (Figure 4b, 5). These embryos did not show any of the 3 characteristic cavities for this stage. In only a few null embryos an unusually small yolk sac space was visible.

**Figure 6:**
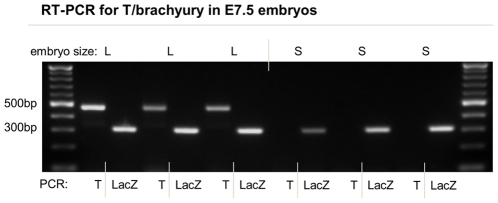
Deletion of *PTBP1* leads to the absence of *T/brachyury*. The figure compares RT PCR results from normal and mutant embryos for the presence of a gastrulation marker and a control. RNA purified from large (L) and small (S) embryos was used as template for reverse transcriptase (RT) PCR amplification of the primitive streak marker *T/brachyury* and of a LacZ control to ensure successful RNA purification. None of the small PTBP1 KO homozygous embryos showed the presence of T mRNA while positive for the control RT PCR.

In normal mouse embryos gastrulation begins at ∼E6.5 with the formation of the primitive streak in the posterior side of the hollowed epiblast. Cells separate into the 3 germ layers. Most null embryos were so malformed that early cell types like ectoderm and endoderm could not be discerned (Figure 4b, 5). Only very few homozygous embryos showed partial separation of cell layers like KO_1_ in Figure 4b. To further investigate the molecular underpinning of development in the null mutants, we performed RT PCR for *T/brachyury* on mutant versus control embryos. T is an embryonic transcription factor expressed in the primitive streak during gastrulation. *T* was consistently detected in normal embryos but absent in all small mutant embryos while a control RT PCR was positive for all samples (Figure 6).

We interpret the absence of cavities, the predominant lack of germ layers, and the failure to activate the gastrulation marker *T* as an indication that *PTBP1* null embryo at E7.5, although viable, are no longer following the normal developmental programme.

### The systemic, homozygous ablation of *PTBP1* is embryonically lethal before E12

We determined the genotype of embryos born from heterozygous parents using whole embryo, yolk sac, or tail clip PCR depending on the size of the animal. In addition, we distinguished between embryos expressing *PTBP1* from the null mutants using immunofluorescence on cryosections. The results, summarised in Table 1, show that homozygous mutant embryos were viable during the first days following implantation but were no longer detected at around mid-gestation. At E12, E16, and the time of weaning, no homozygous animals could be detected by PCR. At the former time points, small deciduas often contain degraded material at their centre that can be neither genotyped nor sectioned. Hence, it appears that after diverging from normal development shortly after implantation, the lack of *PTBP1* becomes lethal shortly after E8.5 and before E12.

### No compensation from PTBP2 or Rod1 in *PTBP1* KO embryos

**Figure 7:**
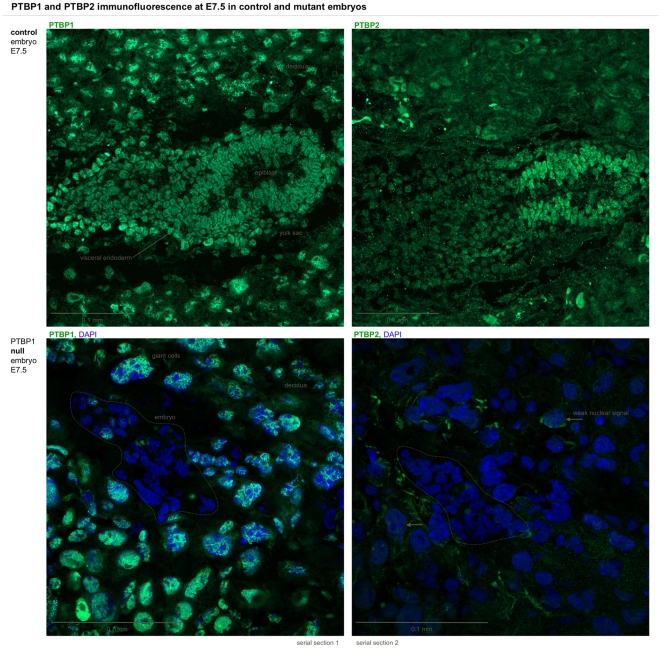
PTBP1 expression in normal and mutant embryos. Comparison of immunofluorescence from PTBP1 and its paralogue PTBP2 in control and PTBP1 KO embryos at E7.5. The top row shows serial cryosections of a normally sized control embryo stained for PTBP1 (left) and PTBP2 (right). PTBP1 was expressed in most embryonic cells, notably the visceral endoderm and the epiblast, comparable to the LacZ reporter staining in Figure 2. A strong PTBP1 signal was also observed in decidual nuclei. PTBP2 in controls was mostly expressed in the epiblast and absent from most decidual nuclei. PTBP1 null embryos (bottom) showed no PTBP1 or PTBP2 signal, displayed here merged with the DAPI signal to reveal the embryo (dashed line). Again most cells surrounding the embryo displayed a nuclear PTBP1 signal while only very few showed a weak nuclear PTBP2 signal (arrow).

PTBP1 suppresses the expression of PTBP2, which is 70% identical in amino acid sequence and fulfils similar functions. In adult tissues, downregulation of PTBP1 often causes upregulation of PTBP2. We were therefore asking ourselves whether a similar compensation could be at work in the *PTBP1* knockout embryos. Control embryos expressed both paralogues, albeit with a different distribution. PTBP1, apart from surrounding decidual cells was highly expressed in the visceral endoderm and the epiblast. This is in agreement with the expression pattern revealed with the LacZ reporter assay (Figure 2). In E7.5 embryos, the strongest PTBP2 fluorescence was found in the epiblast only. However, null mutants showed neither PTBP1 nor PTBP2 immunofluorescent signals at E7.5 (Figure 7). We also looked at whether the 3^rd^, lesser known paralogue of the PTBP group, Rod1, is upregulated in PTBP1 null embryo. Using RNA extracts from E7.5 embryos, we performed RT PCR and did not detect any amplification in the knockout or the control embryos (Figure S5).

We conclude that mutant embryos are not able to compensate for the ablation of *PTBP1* with the upregulation of *PTBP2 or Rod1*. PTBP1 suppresses PTBP2 by exclusion of an essential exon and the upregulation of PTBP2 after reduction of PTBP1 requires an active PTBP2 promoter. It may be that the *PTBP1* knockout in embryos does not cause upregulation of PTBP2 because the cells are so fundamentally perturbed that the *PTBP2* gene is never activated.

### Reduced proliferation and apoptosis in mutant embryos

**Figure 8:**
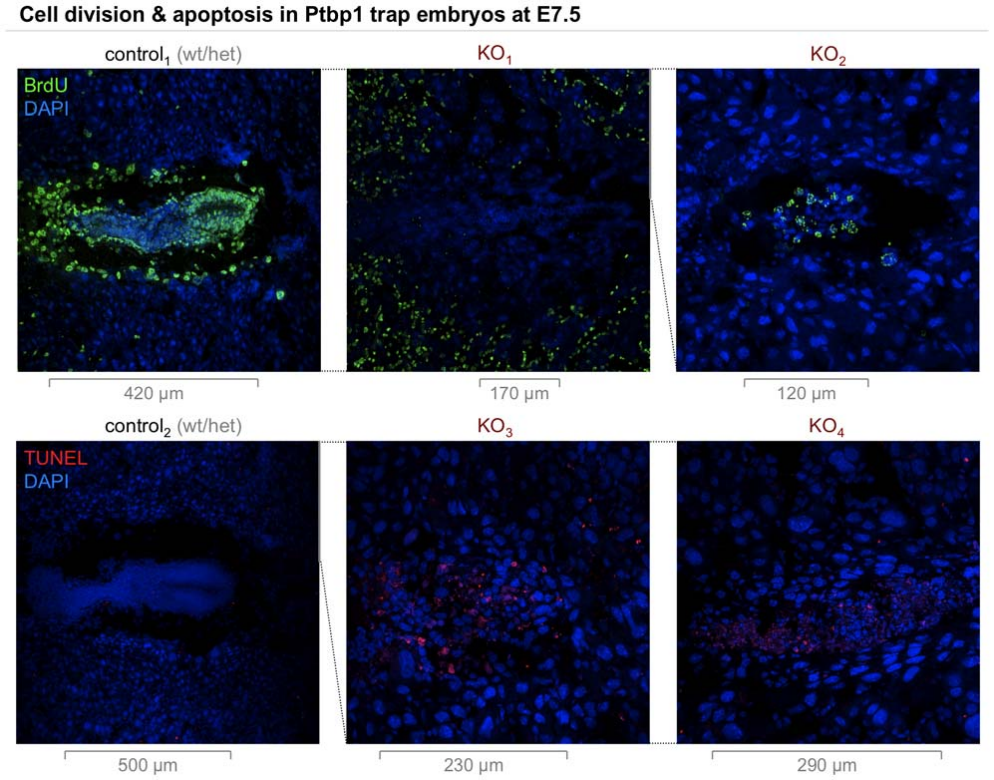
Cell division & apoptosis in mutant embryos. The figure shows control and Ptbp1 null embryo sections at E7.5 immunolabelled for BrdU (top row) and processed by TUNEL (bottom row). The former is a cell division assay measuring the integration of the nucleotide analogue bromodeoxyuridine (BrdU). The latter is an apoptosis assay based on terminal deoxynucleotidyl transferase dUTP nick end labelling (TUNEL). Control embryos (1^st^ column) showed strong BrdU signal especially in the epiblast, the yolk sac area, and the ectoplacental cone while being negative for TUNEL. Heterozygous decidua were typically BrdU-labelled most strongly in the periphery, visible only in KO_1_ and cropped in control_1_ and KO_2_. Homozygous mutant embryos showed less (KO_1_) to no BrdU labelling (KO_2_) varying with embryo and section. Most null mutants analysed showed some degree of TUNEL (exemplified by KO_3_ and KO_4_). The longest diameter of the embryo is indicated below each panel and the relative sizes of the panels are indicated with dashed lines.

To take a more detailed look at cell proliferation in embryos lacking Ptbp1 versus those containing either 1 or 2 normal alleles, we performed BrdU and TUNEL assays (Figure 8). As estimated by BrdU incorporation into newly synthesised DNA, cell division was high throughout the control E7.5 embryos analysed, with especially strong signal coming from the epiblast, the visceral endoderm, the area of the yolk sac, and the ectoplacental cone. Interestingly, the epiblast and the endoderm were also labelled strongly using Ptbp antibodies (Figure 7). There appears to be a correlation between the expression levels of Ptbps and cell division in embryos. Also, null mutants clearly synthesise less DNA.

We detected no TUNEL in control embryos while large parts of the knockout embryos showed DNA fragmentation, indicative of apoptosis (Figure 8, bottom). It appears that after about a week of gestation homozygous Ptbp1 null cells are beginning to apoptose.

## Discussion

### PTBP1 is necessary for early post-implantation development

We generated a new, multi-purpose *PTBP1* allele which was used, in its first stage, to analyse the effect of a systemic knockout. Dissecting pregnancies at several stages, we identified 2 populations of embryos - one with the expected size for the stage of embryonic development and another of smaller size. The difference between the populations was visible as early as E6.5 and widened until the degradation of the small implantation set in after E8.5. Small embryos did not grow significantly after implantation. Genotyping and immunostaining of both embryo populations for *PTBP1* lead us to identify the small population as the homozygous knockout of *PTBP1*. We therefore conclude that *PTBP1* is essential for cell division after implantation, which, unlike earlier mitosis, is associated with significant increase in the cell mass of the embryo.

After characterising the small embryos as homozygous KOs, we analyzed in detail the histology of null embryos at E7.5 and observed that most mutant embryos were not able to commence cavitation or form the 3 germ layers. They also were not able to induce expression of *T/brachyury*, a transcription factor required for the formation of the primitive streak early in gastrulation. This indicates that *PTBP1* is necessary to go through the early post-implantational processes, involving the formation of the egg cylinder and gastrulation.

Null embryos also showed malformed yolk sac and placental structures, often with no discernible yolk sac cavity, an unusually thickened, eosinophilic membrane and giant cells not in their typical position between the Reichert basement membrane and the decidua. A properly formed yolk sac together with early placental structures supplies the embryo with nutrients and enables signalling between the embryo and the mother. Since these structures were malformed in *PTBP1* knockouts, mutant embryos may have been starved and unable to properly communicate with the surrounding maternal tissue. In all likelihood, restricted supply and decreased capacity to divide are compounding developmental problems of the embryos lacking *PTBP1* but further experiments would be required to distinguish between defects in the epiblast and the extra-embryonic supportive structures.

We have observed that *PTBP1* null embryos are able to implant but then remain small, which was not seen in a recent study analysing the effect of mouse *PTBP1* null mutations in embryos and embryonic stem cells [39]. That study found that PTBP1 is expressed both in the trophectoderm and the inner cell mass of the blastocyst and that no stem cell lines could be established from homozygous *PTBP1* knockout blastocyst unlike from those of the other genotypes. However, *PTBP1* null stem cells could be generated by viral insertion of a Cre-expressing construct. The resulting mouse ESCs showed similar levels of *Oct4* and *Sox2* but slightly decreased *Nanog* and *Rex1*, all markers of the undifferentiated state. This indicates that in these cells a degree of pluripotency could be maintained even in the absence of *PTBP1*. However, ESCs without PTBP1 proliferated 8–12x more slowly than heterozygous controls due to delayed G_2_/M progression. These observations fit very well with our data on the increasing size gap between null and control embryos after implantation. Nevertheless, in that study no homozygous mutants were observed at E6.5 and after. Here, on the contrary, we detected the very small homozygous embryos after implantation and analysed their structure. *PTBP1* also emerged as one of the strongest hits from a genome-scale RNAi screen for regulators of *Oct4* [40]. esiRNA targeted against *PTBP1* leads to ∼50% decrease in *Oct4* as well as >60% decrease in *Nanog* transcript levels. This correlated with a loss in alkaline phosphatase staining and a flattening of the characteristically spherical ESC colonies. Thus, it appears that although *PTBP1* is not an absolute requirement for stemness, it does have a strong positive effect on the pluripotency of embryonic stem cells.

### RNA-binding proteins in embryonic development

The data detailed above [39] together with the present findings firmly establish *PTBP1* as a factor essential for early embryonic development, in particular due to its necessity for speedy cell division. *PTBP1* joins ranks with several other RNA-binding proteins that cause severe defects in early embryonic development. For example, the knockout of *SSB*, which like *PTBP1* contains RRMs, completely prevents implantation and the generation of stem cells from blastocysts [41]. Null embryos for *ELAVL1*, composed of 3 RRMs and a SPOC domain, implant and begin gastrulation but then die between E10.5 and E14.5 due to placental malformation [42]. Another RRM-containing protein, TIAR, has a more specific defect, as it is required for survival of primordial germ cells and the formation of spermatogonia and oogonia, probably due to its effect on cell division [43].

### Splicing or stabilisation

At the current state of the analysis it is not clear which molecular function of PTBP1 is responsible for the drastic developmental defects of its null embryos. The aforementioned stabilising effect of PTBP1 on CU-tract containing mRNAs may be required to provide enough messenger for protein synthesis when it is upregulated in the volume growth phase of the embryo after implantation. On the other hand, the control PTBP1 exerts on the splicing pattern of embryonic cells may be crucial for the proliferation of the early embryo. Part of the differentiation process is the divergence of splicing programmes. Since the pre-implantation embryo contains only few different tissues, splicing patterns are probably similar. After implantation, many different tissues arise and may require *PTBP1* to control exon selection. Further experiments, for example CLIP [44] to identify the targets of PTBP1, mRNA decay assays to test for potential stabilising effects and splice form-sensitive microarrays may clarify these points. Possibly, even the not yet well-established role of *PTBP1* in binding gene promoter regions may play a role. The main challenge for these studies will be the very low amount of homozygous mutant material that can be obtained.

### Outlook

The Ptbp1 mutant mouse generated and analysed here demonstrates how the absence of an RNA-binding protein, involved in splicing and RNA stability among other functions, can completely halt embryonic development. It also shows that the paralogues Ptbp2 and Rod1 are not able to compensate for the loss of Ptbp1. Embryonic cells lacking Ptbp1 can divide in principle but do so at a much slower rate. In addition and possibly linked to the former, Ptbp1 null embryos have problems with correct differentiation.

With the Yoshida lab allele (loxP-framed exon 1) and our multi-purpose allele (FRT-framed stop/detection cassette and loxP-framed exon 3–7) two new PTBP1 alleles have been generated which can be combined with a wide variety of Cre strains for conditional deletion. The allele used in this study, in its first stage before combination with a FLP recombinase, could also be exploited to detect the activity of the *PTBP1* locus in various tissues during ontogeny as exemplified by our data for embryonic developmental stage E12.5 and E16.5. Considering the strong interest in embryonic stem cells and regulation of splicing patterns these *PTBP1* alleles will be an important tool to further dissect the multiple functions of this nucleic acid-binding protein. More specifically, the second stage of the multi-purpose allele after recombinase conversion could be used for the conditional knockout of *PTBP1* in pancreatic β-cells only [45] in order to test the role of this gene in insulin granule biogenesis and diabetes [18], [19], [46].

## Materials and Methods

### Generation of a PTBP1 multi-purpose mutant mouse allele

The PTBP1 genomic sequence used for the construction of the mutant allele was based on the male C57BL/6J bacterial artificial chromosome RPCI24-368K20, obtained from the CHORI's BACPAC. Part of *PTBP1* was first subcloned and later modified in several steps via Red/ET recombination (Gene Bridges) in *E. coli* bacteria. The final targeting construct was linearised, electroporated into R1/E 129 mouse stem cells grown on feeders and selected using HygB. The correct insertion of the targeting construct into the genome was subsequently confirmed by Southern blot on genomic DNA and junction PCR over the homologous recombination arm. The full sequence of the targeting construct used to modify the PTBP1 locus can be found in the nucleotide databases like GenBank under the identifier HM588137 (http://www.ncbi.nlm.nih.gov/nuccore/307557308).

### Generation of PTBP1 KO mice

The verified stem cell clones were selected for morula injection with a germline transmission rate per embryo of 0/50 and 5/80 for the 2 clones respectively. Chimeras were inbred with C57BL/6J for 5–8 generations before being intercrossed and analysed. All animal experiments were conducted in a licensed animal facility in accordance with the *German Animal Welfare Act*, following the guidelines of the *European Convention for the Protection of Vertebrate Animals Used for Experimental and Other Scientific Purposes* and approved by the Regional Council (24-9168.24-1-2003-2).

### Genotyping

Adult PTBP1 mutant mice were genotyped from a tail clip after proteinase K digestion using 2 primer pairs. Pair 1 (forward: 5′-CTGTAGCGGCTGATGTTGAA, reverse: ATGGCGATTACCGTTGATGT, product: 299 bp) detects the LacZ reporter gene and differentiates between wild-type (wt) and heterozygous/homozygous mice. Pair 2 (CCTCATTTGCTGTCCTGGTT, CCCTGGTGTCCTGTCATCTT, product 208 bp) binds to intronic sequences around the insertion site of the stop and detection cassette and differentiates between hom and wt/het genotypes.

Due to the small amount of early embryonic tissue, the entire embryo were used for genotyping at gestational stages before E9. The embryonic tissue was carefully dissected to avoid contamination by tissue from the mother and then genotyped as above.

### Embryo cryosections and X-gal staining

Gestation was timed according to the morning-after plug check. Mice were sacrificed after isoflurane narcosis by cervical dislocation. Uteri were dissected in ice-cold phosphate-buffered saline. Isolated deciduas or embryos were immersed in 30% (m/v) sucrose in phosphate-buffered saline (PBS) for 30 min to avoid cryoartefacts. Samples were then embedded in Tissue-Tek (Sakura 4583), frozen using liquid nitrogen, cut into 10 µm thick sections and mounted. Samples were fixed in PBS with 0.2% (v/v) glutaraldehyde, 5 mM EGTA and 0.1 M MgCl_2_ at 4°C for 10 min. Sections were then washed 3x 5 min in LacZ wash buffer (PBS with 0.01% sodium deoxycholate, 0.02% NP40 and 2 mM MgCl_2_) and incubated overnight in LacZ staining buffer containing 2.45 mM 5-bromo-4-chloro-3-indoxy-β-D-galactopyranoside (X-gal). The staining buffer was prepared by diluting an X-gal stock (1g X-gal in 40 ml dimethylformamide) 1∶25 in LacZ wash buffer and adding 5 mM K-ferrocyanide and 5 mM K-ferricyanide. After removal of the staining solution and 3 PBS washes, the X-gal staining was allowed to intensify in PBS at 4°C before mounting and bright field imaging.

### Histology and immunohistochemistry

The entire deciduas were fixed in 4% paraformaldehyde overnight at 4°C, dehydrated and embedded in paraffin. 5 µm sections were cut, deparaffinised and stained with hematoxylin and eosin. Serial sections were used for immunodetection of PTBP1. Antigen retrieval was performed by treatment of the slides with Tris-EDTA buffer (10 mM Tris Base, 1 mM EDTA Solution, 0.05% v/v Tween 20, pH 9.0) for 40 min. at 95°C. The sections were then incubated overnight at 4°C with the anti-PTBP1 antibody (Invitrogen, prev. Zymed, #32-4800) or the anti-collagen IV antibody both diluted 1∶200 in PBS. Detection of the primary antibody was achieved by incubating the sections with a Cy3-conjugated goat anti-mouse IgG (Jackson Immuno Research Laboratories) diluted 1∶500 in PBS at room temperature for 45 min. Sections were finally mounted with Mowiol (Biovalley) containing DAPI at 10 µg/ml and imaged with a fluorescence microscope equipped with DAPI and CY3 filters.

For immunolabelling of embryo cryosections, the decidua was dissected, embedded in TissueTek OCT and then frozen on dry ice. 10 µm sections were cut and mounted on glass slides. Sections were washed with PBS and then blocked by incubation in goat serum buffer (16% v/v goat serum, 20 mM phosphate buffer at pH 7.4, 0.45 M NaCl, 0.3% v/v Triton-X-100) at room temperature for 1 h. Samples were then incubated with the anti-PTBP1 or anti-PTBP2 antibody (Abnova H00058155-A01) diluted 1∶200 in goat serum buffer at 4°C overnight. Primary antibodies were detected by incubation with goat anti-mouse IgG_1_-Alexa488 (Invitrogen) diluted 1∶200 in goat serum buffer and DAPI at 5 µg/ml at room temperature for 45 min.

### Reverse transcriptase PCR

Two E7.5 litters from a heterozygous intercross were dissected in ice-cold PBS, washed 2x in cold PBS to reduce the risk of maternal contamination, and extracted using TRIzol (Invitrogen). RNA was reverse transcribed using SuperScript II (Invitrogen). RT PCR was performed using *T/brachyury* primers (forward primer: TCCCAATGGGGGTGGCTTGTTCCTG, reverse primer: ACCAGAAGACGAGGACGTGGCA; expected product: 474 bp) and a different set of LacZ primers (forward: CTGGCTACCGGCGATGAGCG, reverse: GATCAGCGGGCGCGTCTCTC, product; 310 bp) and Platinum Taq (Invitrogen). PCR products were analysed on agarose gels.

### BrdU assay

A total of 0.6 mg BrdU/10 g body weight in sterile PBS was injected into the stomach cavity (IP) of pregnant mice at day 7. Mice were sacrificed and embryos were dissected out of uterus in cold PBS 3 hours later. Embryos were immersed in 9%, 18% and 30% w/v sucrose in PBS at 4°C before being embedded in Tissue-Tek and frozen at −80°C. Blocks were cryosectioned at 8 µm thickness.

Serial sections were fixed in 4% w/v PFA at 4°C for 30 minutes and permeabilised in PBS and 0.3% v/v Triton X-100 at RT for 1 hour. Sections were washed 3x 5 minutes with PBS at RT. 2 mol/L HCl treatment was applied to all sections at RT for 25 minutes. Sections were washed 3x 5 minutes with PBS and blocked with 10% v/v goat serum and 0.3% Triton X-100 in PBS at RT for 1 hour. Afterwards, they were incubated overnight with primary antibody (rabbit anti-BrdU, abcam ab6326, diluted 1∶500 in blocking buffer). Sections were washed 3x 5 minutes in PBS at RT and incubated with secondary antibody (AlexaF 555 goat anti-rabbit, Molecular Probes, diluted 1∶500 in blocking buffer) and DAPI (5 ng/µl) at RT for 1 hour. Sections were washed 3x 5 minutes in PBS at RT and mounted with Mowiol. Images were made using a Zeiss LSM 510 confocal microscope.

### TUNEL assay

Samples were dissected as described above for the BrdU assay. Serial sections were fixed in 4% w/v PFA at 4°C for 20 minutes and washed with PBS at RT for 30 minutes. Sections were permeabilised in 0.1% v/v Triton X-100 for 2 minutes on ice. Sections were incubated in staining solution (450 µl dUTP-TMR labelling solution +50 µl terminal transferase solution both from the Roche in situ cell death detection kit #12156792910 and added DAPI at 5 ng/µl) at 37°C for 60 minutes. Sections were briefly rinsed 3x with PBS and mounted in Mowiol. Images were made using a Zeiss LSM 510 confocal microscope.

## Supporting Information

Figure S1 **Construction of the PTBP1 multi-purpose allele.** The figure shows the Red/ET homologous recombination strategy used to construct the *PTBP1* multi-purpose allele. A) The genomic sequence was derived from a bacterial artificial chromosome (BAC) and subcloned into a more easily handled bacterial vector. First, the lower loxP site was inserted, selected for and removed via transient transfection with a Cre-expressing plasmid. Second, the FRT-framed stop cassette and upper loxP site were inserted and selected for. B) The verified targeting construct was cleaved from the plasmid, electroporated into mouse embryonic stem cells and selected for. (ori - origin of replication; sA - splice acceptor; IRES - internal ribosome entry site; b-galactosidase/hygromycin-B fusion; STOP codon; polyA signal; recombinase recognition sites: FRT - Flp recombinase; loxP - Cre recombinase).
(TIFF)

Figure S2 **Verification of the effectiveness of the Ptbp1 stop cassette.** Transcription stop cassettes, as the one used in this study, have on occasions been observed to be breached, resulting in the synthesis of some full-length mRNA [PMIDs 8978605, 9039657, 16575173]. To verify the effectiveness of the Ptbp1 null allele employed here, we designed primers for the 3′ end of Ptbp1 and performed RT PCR on small homozygous and large heterozygous/wild type embryos at E7.5. We observed that the 3′ UTR of Ptbp1 could be amplified in all large embryos, while most homozygous embryos showed no product from the 3′ Ptbp1 primers. In the few cases (red) were a faint signal could be observed, null embryos were not well segregated from the surrounding maternal tissue. The signal is likely to have arisen from a small number of maternal cells that could not be removed during the dissection. cDNA quality was confirmed using a tubulin α1b primer. Primer sequences: Tuba1b cagtgttcgtagacctggaacc & ctgtggaaaaccaagaagccctg, product 226 bp; Ptbp1 exon 12/13 to exon 14 acctctccaacatcccgccct & gcaggtggtggttctcgccc, product 198 bp (stop cassette in intron 2).
(TIFF)

Figure S3 **Whole-mount LacZ staining of embryos.** Complementing the LacZ staining on sections (Figure 2), this figure shows whole-mount LacZ staining at E12.5 and E16.5. The X-gal signal seen here is superficial since the dye could not penetrate deeply into embryos at these stages. However, due to the thickness of the sample, areas with weak expression can be recognised. For example at E16.5 the skin appeared unstained in the sections while from the whole-mount it became clear that the reporter was expressed also there.
(TIFF)

Figure S4 **Collagen separate channels.** This figure shows the same embryos as in Figure 5b and an additional PTBP1 null mutant (3rd column). Confocal fluorescence from DAPI and collagen 4 antibodies is displayed separately. In addition, a hematoxylin and eosin stained serial section is shown for each of the 3 embryos. The 2nd row emphasises the aberrant localisation of collagen 4 in embryos lacking PTBP1 versus those expressing normal levels.
(TIFF)

Figure S5 **Rod1 mRNA is not detected in E7.5 embryos.** Rod1 is a nucleic acid binding protein, paralogous to Ptbp1 and Ptbp2, and known in yeast to suppress differentiation. To test whether Rod1 is upregulated in response to the absence of Ptbp1/2, we performed RT PCR on small homozygous and large heterozygous/wild type embryos 7.5 days of age. Neither the null mutants nor the control embryos appeared to express Rod1 mRNA. Efficiency of the Rod1 amplification was confirmed with a Rod1 cDNA positive control (left); the expected product size is 423/448 bp depending on the Rod1 splice variant. cDNA synthesis was confirmed using a tubulin α1b control primer pair (product size 226 bp). We tested 3 other Rod1 primer pairs also without amplification. This gel shows the products of Tuba1b primers cagtgttcgtagacctggaacc & ctgtggaaaaccaagaagccctg as well as Rod1 primers gcggtgagcccgtcaatccc & tctcggtgattggaatactggat.
(TIFF)
