# Supplementary figures and images for: PTBP1 Is Required for Embryonic Development before Gastrulation
Source: PLoS One. 2011 Feb 17;6(2):e16992. doi: 10.1371/journal.pone.0016992 (PMC3040740; doi:10.1371/journal.pone.0016992)

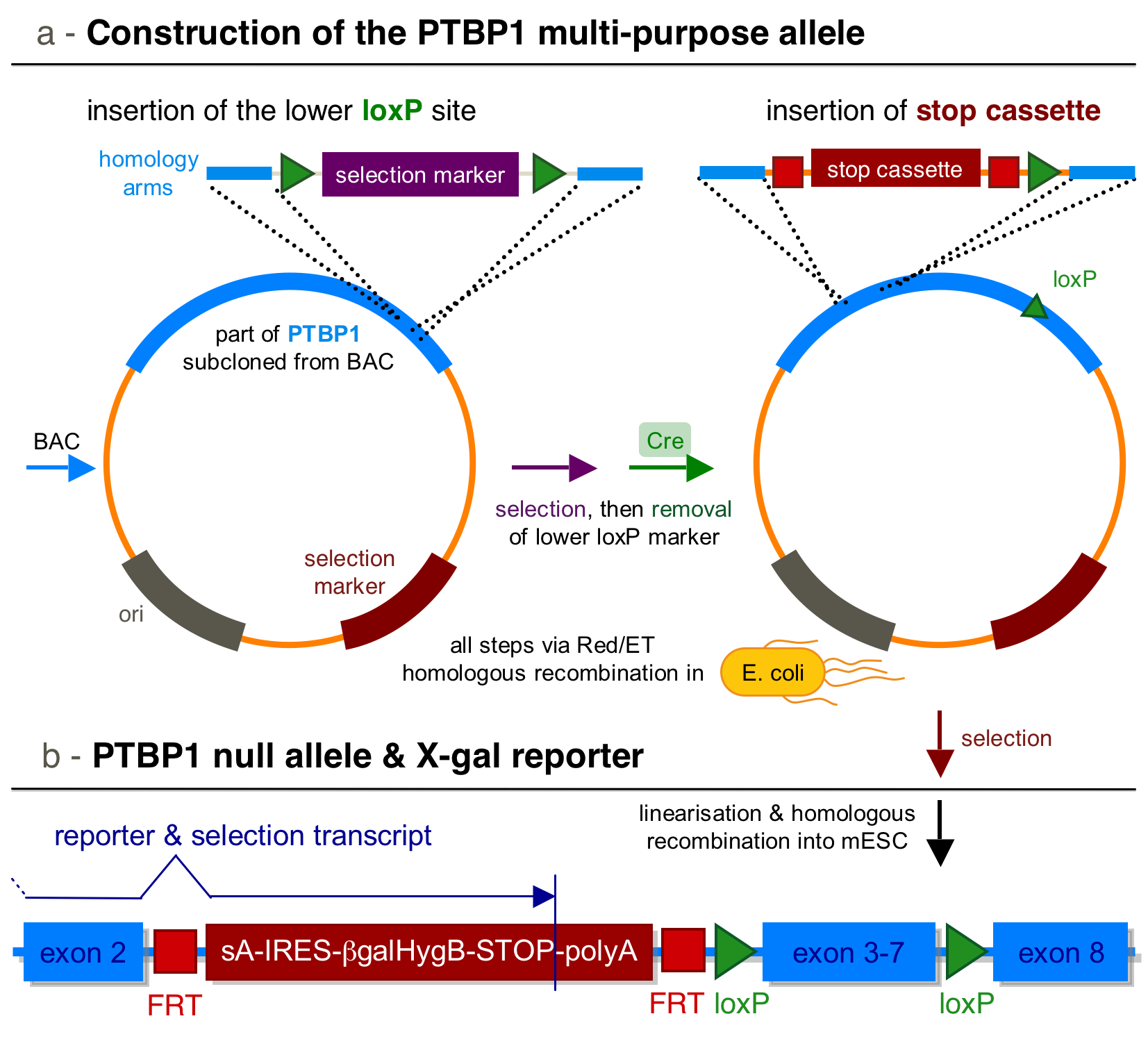

Supplement: Figure S1 — Construction of the PTBP1 multi-purpose allele. The figure shows the Red/ET homologous recombination strategy used to construct the PTBP1 multi-purpose allele. A) The genomic sequence was derived from a bacterial artificial chromosome (BAC) and subcloned into a more easily handled bacterial vector. First, the lower loxP site was inserted, selected for and removed via transient transfection with a Cre-expressing plasmid. Second, the FRT-framed stop cassette and upper loxP site were inserted and selected for. B) The verified targeting construct was cleaved from the plasmid, electroporated into mouse embryonic stem cells and selected for. (ori - origin of replication; sA - splice acceptor; IRES - internal ribosome entry site; b-galactosidase/hygromycin-B fusion; STOP codon; polyA signal; recombinase recognition sites: FRT - Flp recombinase; loxP - Cre recombinase). (TIFF) [file pone.0016992.s001.tiff]

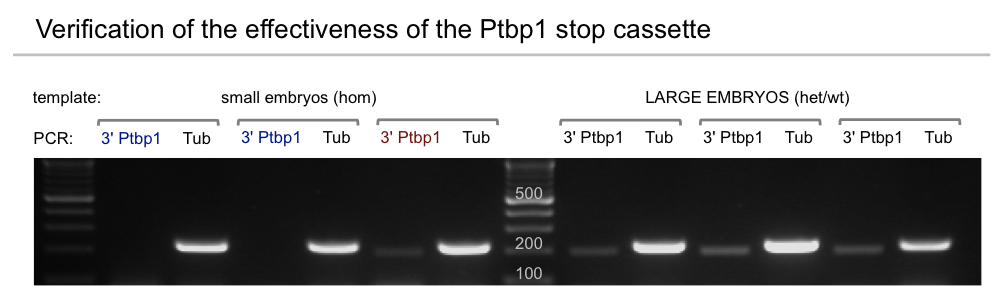

Supplement: Figure S2 — Verification of the effectiveness of the Ptbp1 stop cassette. Transcription stop cassettes, as the one used in this study, have on occasions been observed to be breached, resulting in the synthesis of some full-length mRNA [PMIDs 8978605, 9039657, 16575173]. To verify the effectiveness of the Ptbp1 null allele employed here, we designed primers for the 3′ end of Ptbp1 and performed RT PCR on small homozygous and large heterozygous/wild type embryos at E7.5. We observed that the 3′ UTR of Ptbp1 could be amplified in all large embryos, while most homozygous embryos showed no product from the 3′ Ptbp1 primers. In the few cases (red) were a faint signal could be observed, null embryos were not well segregated from the surrounding maternal tissue. The signal is likely to have arisen from a small number of maternal cells that could not be removed during the dissection. cDNA quality was confirmed using a tubulin α1b primer. Primer sequences: Tuba1b cagtgttcgtagacctggaacc & ctgtggaaaaccaagaagccctg, product 226 bp; Ptbp1 exon 12/13 to exon 14 acctctccaacatcccgccct & gcaggtggtggttctcgccc, product 198 bp (stop cassette in intron 2). (TIFF) [file pone.0016992.s002.tiff]

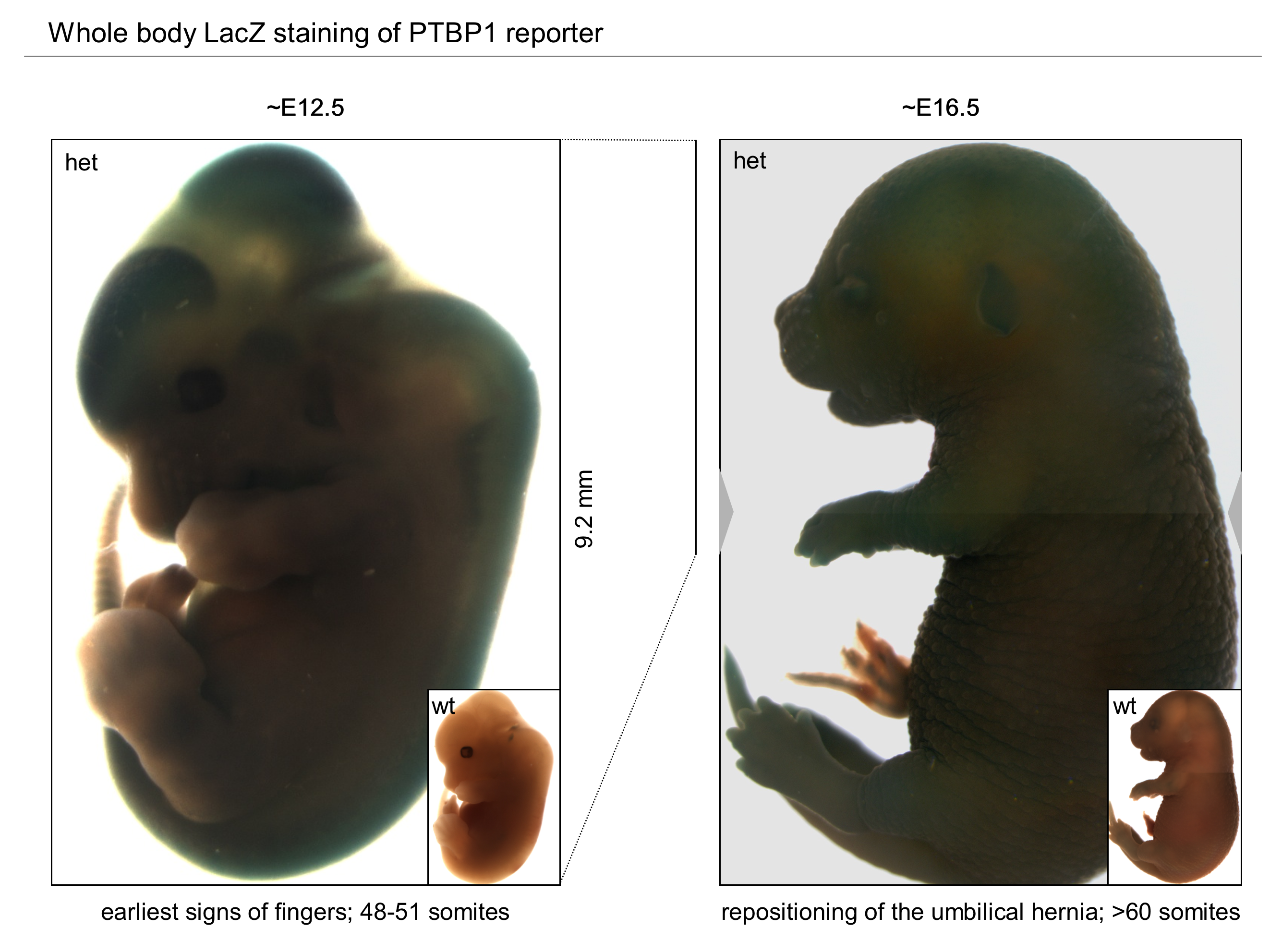

Supplement: Figure S3 — Whole-mount LacZ staining of embryos. Complementing the LacZ staining on sections (Figure 2), this figure shows whole-mount LacZ staining at E12.5 and E16.5. The X-gal signal seen here is superficial since the dye could not penetrate deeply into embryos at these stages. However, due to the thickness of the sample, areas with weak expression can be recognised. For example at E16.5 the skin appeared unstained in the sections while from the whole-mount it became clear that the reporter was expressed also there. (TIFF) [file pone.0016992.s003.tiff]

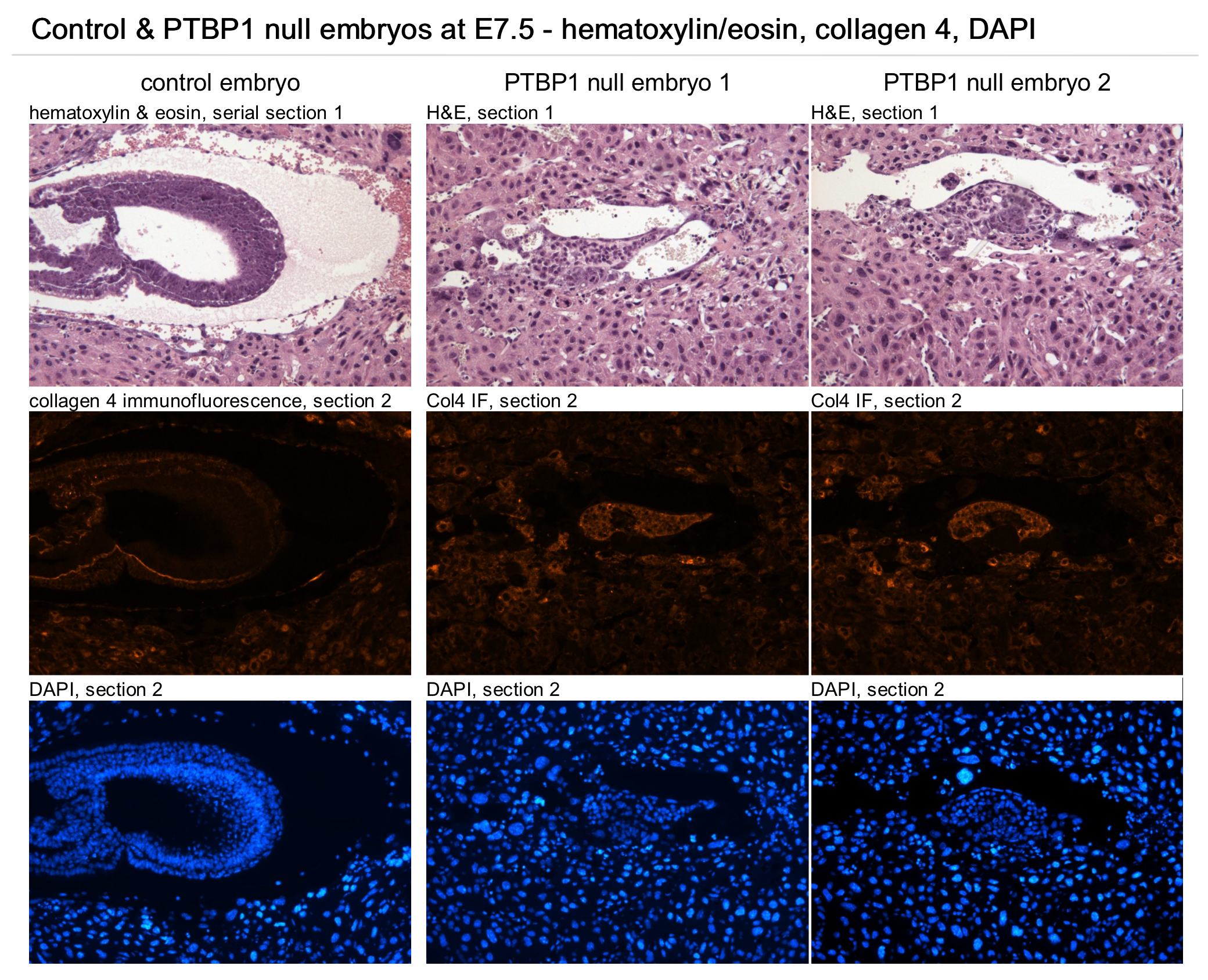

Supplement: Figure S4 — Collagen separate channels. This figure shows the same embryos as in Figure 5b and an additional PTBP1 null mutant (3rd column). Confocal fluorescence from DAPI and collagen 4 antibodies is displayed separately. In addition, a hematoxylin and eosin stained serial section is shown for each of the 3 embryos. The 2nd row emphasises the aberrant localisation of collagen 4 in embryos lacking PTBP1 versus those expressing normal levels. (TIFF) [file pone.0016992.s004.tiff]

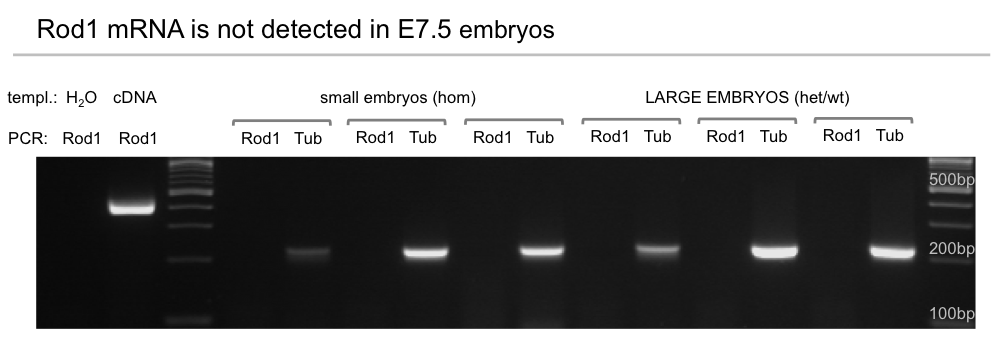

Supplement: Figure S5 — Rod1 mRNA is not detected in E7.5 embryos. Rod1 is a nucleic acid binding protein, paralogous to Ptbp1 and Ptbp2, and known in yeast to suppress differentiation. To test whether Rod1 is upregulated in response to the absence of Ptbp1/2, we performed RT PCR on small homozygous and large heterozygous/wild type embryos 7.5 days of age. Neither the null mutants nor the control embryos appeared to express Rod1 mRNA. Efficiency of the Rod1 amplification was confirmed with a Rod1 cDNA positive control (left); the expected product size is 423/448 bp depending on the Rod1 splice variant. cDNA synthesis was confirmed using a tubulin α1b control primer pair (product size 226 bp). We tested 3 other Rod1 primer pairs also without amplification. This gel shows the products of Tuba1b primers cagtgttcgtagacctggaacc & ctgtggaaaaccaagaagccctg as well as Rod1 primers gcggtgagcccgtcaatccc & tctcggtgattggaatactggat. (TIFF) [file pone.0016992.s005.tiff]
